# Electrochemical Biosensing of L-DOPA Using Tyrosinase Immobilized on Carboxymethyl Starch-*Graft*-Polyaniline@MWCNTs Nanocomposite

**DOI:** 10.3390/bios13050562

**Published:** 2023-05-21

**Authors:** Fahimeh Mollamohammadi, Hassan Faridnouri, Ehsan Nazarzadeh Zare

**Affiliations:** 1 School of Biology, Damghan University, Damghan 36716-45667, Iran; fmollamohammadi33009@gmail.com; 2 School of Chemistry, Damghan University, Damghan 36716-45667, Iran

**Keywords:** carboxymethyl starch, polyaniline, MWCNTs, tyrosinase, electrical nanocomposite

## Abstract

The electrochemical behavior of the immobilized tyrosinase (Tyrase) on a modified glassy carbon electrode with carboxymethyl starch-*graft*-polyaniline/multi-walled carbon nanotubes nanocomposite (CMS-*g*-PANI@MWCNTs) was investigated. The molecular properties of CMS-*g*-PANI@MWCNTs nanocomposite and its morphological characterization were examined by Fourier transform infrared spectroscopy (FTIR), X-ray diffraction (XRD), and field emission scanning electron microscopy (FESEM). A simple drop-casting method was employed to immobilize Tyrase on the CMS-*g*-PANI@MWCNTs nanocomposite. In the cyclic voltammogram (CV), a pair of redox peaks were observed at the potentials of +0.25 to −0.1 V and E°’ was equal to 0.1 V and the apparent rate constant of electron transfer (K_s_) was calculated at 0.4 s^−1^. Using differential pulse voltammetry (DPV), the sensitivity and selectivity of the biosensor were investigated. The biosensor exhibits linearity towards catechol and L-dopa in the concentration range of 5–100 and 10–300 μM with a sensitivity of 2.4 and 1.11 μA μΜ^−1^ cm^−2^ and limit of detection (LOD) 25 and 30 μM, respectively. The Michaelis-Menten constant (K_m_) was calculated at 42 μΜ for catechol and 86 μΜ for L-dopa. After 28 working days, the biosensor provided good repeatability and selectivity, and maintained 67% of its stability. The existence of -COO^−^ and -OH groups in carboxymethyl starch, -NH_2_ groups in polyaniline, and high surface-to-volume ratio and electrical conductivity of multi-walled carbon nanotubes in the CMS-*g*-PANI@MWCNTs nanocomposite cause good Tyrase immobilization on the surface of the electrode.

## 1. Introduction

Enzyme-based biosensors are widely used in electrochemical technique studies due to the enzymes’ biocatalytic activity and enzyme-analyte specific and selective binding ability [1,2]. Electrochemical enzymatic biosensors (EEBs) measure electrical signals caused by electron exchange in the electrode-enzyme common surface, which is the result of target analytes oxidation or reduction by the enzyme, and it correlated to the substrate concentration in the solution sample [3]. The advantages of combining EEBs with wireless technologies as portable and miniature devices are simplicity, low-cost fabrication, high sensitivity, and rapid response, and have turned them into accessible tools in all application fields of medical diagnosis and health, environmental monitoring, and specific analyte detection [3,4]. In this type of biosensor, the use of free enzymes has no economic justification and they are less stable against environmental changes [5,6]. Therefore, the selection of suitable retainers for the immobilization of enzymes is very remarkable, because the properties of the matrices have a significant effect on the activity of the immobilized enzyme [7]. With the progress in nanotechnology, nanoscale materials such as nanoparticles, nanotubes, and nanocomposites have been considered immobilizing matrices [8]. Carbon nanotubes (CNTs) are one of the most widely-used nanomaterials in the development of biosensors and multifunctional nanocomposites due to their excellent stability in aqueous and non-aqueous solutions, fast electron transfer, and high mechanical strength [9]. The high surface-to-volume ratio provides a suitable matrix for more immobilization of biomolecules on their surface [10,11,12].

In recent years, inherently conducting polymers, especially polyaniline (PANI), have attracted significant attention for modification of the electrodes [13]. The redox state and electrical features of PANI facilitated the electron tunneling between the active site of the enzyme and the electrode hydrophilic surface, which causes reaction potential measured at the close of the potential of the enzyme’s prosthetic group in the biosensing system [14,15]. Furthermore, PANI has excellent and controllable chemical features, low cost, easy synthesis, and a stable–flexible nanostructure [16]. However, the limitations of using PANI in the preparation of polymer film, molding, and its poor solubility can prevent the effective binding of the enzyme in the biosensing system [17]. The copolymerization of the PANI with a modified biopolymer is a promising method for enhancing the immobilization of enzymes in the biosensors [18]. The biopolymers as the biocompatible matrix can protect the enzyme active sites from the negative effects of the process conditions. The functionalized biopolymers via carboxylic (COOH), hydroxyl (OH), amine (NH_2_) groups, etc. can be used for loading more enzymes in the PANI’s nanocomposites [7,19]. Starch is one of the cost-effective natural polymers that is used in the food industry [20]. The modification of starch with various compounds is one of the methods for improving its properties, such as processability [21]. Carboxylation is a simple method for the formation of a water-soluble polysaccharide [22]. Carboxylated starch or, more precisely, carboxymethyl starch (CMS) is a water-soluble polysaccharide that is widely employed as an additive. The CMS is a biodegradable and non-toxic product that is used as a thickener, binder, and emulsifying agent in various applications [20,21,22]. Decreasing the current transmission in the polyaniline can be compensated via carbon nanotubes after copolymerization [18].

According to our previous work experience [18], it is anticipated that carboxymethyl starch can reduce steric hindrance for better access of the substrate to the enzyme active site by electrostatic binding to the protein shell structure and provide a biocompatible environment similar to biological membranes for the enzyme catalytic activity, and it provides a suitable immobilizing matrix for EEBs through a synergic effect with polyaniline and carbon nanotube.

In the present study, we attempted to make suitable, easy, low-cost, conducting, and biocompatible support for loading and better immobilization of the enzyme via the construction of carboxymethyl starch-*graft*-polyaniline@multi-walled carbon nanotubes nanocomposite (CMS-*g*-PANI@MWCNTs). Considering the sensitivity of detecting phenolic compounds in environmental research and the field of neuroscience, which are the pioneering fields in the production of biosensors, we chose Tyrase to measure the electrochemical parameters of a modified electrode in the presence of the substrates catechol and L-dopa.

## 2. Materials and Methods

### 2.1. Materials

Tyrase (EC 1.14.18.1; activity ≥ 2700 units mg^−1^ of solid, from mushroom), 3,4-dihydroxy-L-phenylalanine (L-DOPA), and 1,2-dihydroxybenzene (pyrocatechol) were acquired from Sigma Co., (Ronkonkoma, NY, USA). Multi-walled carbon nanotubes (MWCNTs) with outside dimensions 6–9 nm were purchased from Nutrino Company (Tehran, Iran). Corn starch was provided by Kimia Eksir Asia Company (Tehran, Iran). Aniline monomer, isopropanol, methanol, sodium hydroxide, chloroacetic acid, hydrogen chloride (HCl 37%), ammonium peroxy disulfate (APS), uric acid (UA), and ascorbic acid (AA) were acquired from Merck Company (Germany). All reagents were used without further purification. A phosphate buffer solution (PBS 0.1 M, pH = 6.8 at 25 ± 2 °C prepared by using K_2_HPO_4_ and KH_2_PO_4_) was used to prepare the electrolyte. All solutions were made ready with distilled water.

### 2.2. Apparatus

CV and differential pulse voltammetry (DPV) were recorded using a galvanostat/potentiostat (RADstat 10, Kianshar Danesh, Tehran, Iran) with a standard three-electrode system including a working electrode (a glassy carbon electrode, GCE, Azarelectrode, Iran, diameter of 2 mm), Ag/AgCl reference electrode (3 M KCl), and counter electrode (a platinum wire, Pt). To compare the interfacial properties of the bare and modified electrode surfaces, electrochemical impedance spectroscopy (EIS) was performed by a device of Autolab electrochemistry with galvanostat/potentiostat/ impedance analysis (Autolab PGSTAT-30, NOVA Saftware, Emmen, The Netherlands). EIS was conducted in the presence of K_3_[Fe(CN)_6_]/K_4_[Fe(CN)_6_] 0.005 M in potassium chloride (KCl) 0.1 M as a redox probe in the frequency range of 0.10 Hz to 50 kHz, amplitude of 5.0 mV. The pH value of all solutions was adjusted using a digital pH meter with an accuracy of ±0.001 (780 pH meter, Metrohm). For surface morphology studies, field emission scanning electron microscopy (FESEM)/EDS (Tescan, MIRA3-XMU) was recorded at room temperature. An Equinox55 spectrometer apparatus (Bruker Optik GmbH, Leipzig, Germany) recorded Fourier transform infrared (FTIR) spectra in the wavenumber range of 4000–400 cm^−1^. The X-ray diffraction patterns (XRD, BrukerD8 Advance, Germany) were recorded using Cu-Kβ radiation in the 2θ range of 5–70° at room temperature.

### 2.3. Sodium Carboxymethyl Starch (CMS) Preparation

Sodium carboxymethyl starch (CMS) was prepared according to our previously-reported method [23], using 1 g of corn starch dissolved in 15 mL isopropanol as a solvent. After 15 min, 1.2 g of sodium hydroxide was added to the vessel containing starch and stirred continuously at 40 °C for 1 h. Monochloroacetic acid (1.36 g) was added, and the stirring continued for another hour. The white CMS precipitate was settled with the increase in the functionalized centers. The centrifuged precipitate was washed with 80% methyl alcohol several times and dried at room temperature for 24 h.

### 2.4. Fabrication of Carboxymethyl Starch-Graft-Polyaniline@ Multi-Walled Carbon Nanotubes (CMS-g-PANI@MWCNTs) Nanocomposite

**Figure 1:**
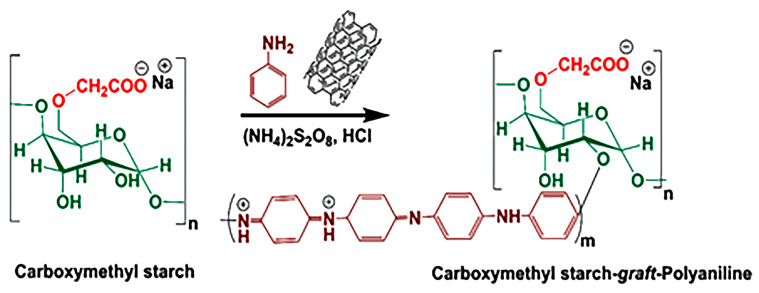
Construction of carboxymethyl starch-*graft*-polyaniline@multi-walled carbon nanotubes (CMS-*g*-PANI@MWCNTs) nanocomposite.

An in situ copolymerization method was used for the fabrication of the CMS-*g*-PANI@MWCNTs nanocomposite as follows: first, CMS powder (1 g) was completely dissolved in 70 mL of distilled water at 50 °C by a magnetic stirrer for 30 min. After cooling the solution to room temperature, 0.15 g of dispersed carbon nanotubes in 30 mL of distilled water were added to the balloon’s contents. The system was initially exposed to nitrogen gas. In another flask, 3 g of ammonium peroxy disulfate initiator was dissolved in 20 mL of distilled water and was added to the vessel contents drop-by-drop at a temperature of 0–5 °C over 15 min. Aniline monomer (2 mL) was then added to the reaction mixture. To complete the copolymerization reaction, the system was left under nitrogen gas for 12 h at ambient temperature. In the following, 50 mL of 2 M hydrochloric acid was added to the reaction balloon for 2 h to form the emeraldine-salt (EM-S, conducting a form of PANI). In the end, the nanocomposite black precipitate was centrifuged in the plastic falcons and washed with methanol and distilled water several times, and then dried at ambient temperature. Figure 1 displays the synthesis pathway reaction of (CMS-*g*-PANI@MWCNTs) nanocomposite.

### 2.5. Enzyme Immobilization on the Modified Electrode

**Figure 2:**
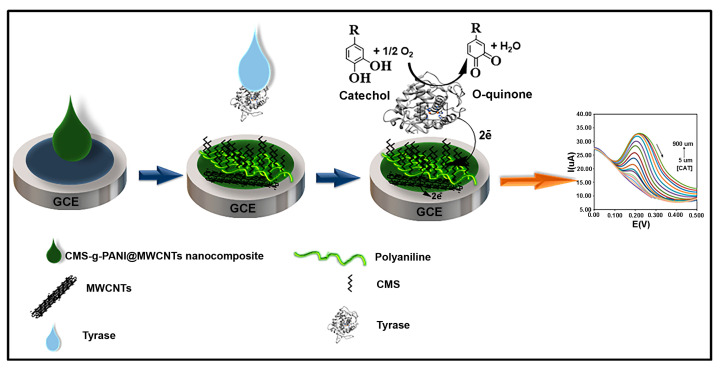
Preparation of the modified electrode.

To prepare the modified electrode, first, the polished and cleaned surface of the bare glassy carbon electrode (GCE) was placed in the potassium phosphate buffer under a voltage of 1.5 V for 2 min to make a hydrophilic electrode surface [24]. 5 μL of prepared nanocomposite solution (1 mg/mL, 1 mg sonicated nanocomposite in 1 mL PBS solvent) was then dropwise placed onto the bare GCE surface (CMS-*g*-PANI@MWCNTs/GCE). After drying the nanocomposite at room temperature, 5 μL of Tyrase (1.25 mg/mL, dissolved in PBS, pH 6.8) was added to the modified GCE surface (CMS-*g*-PANI@MWCNTs/Tyrase/GCE). The modified electrode was kept in a refrigerator at a temperature of 4 °C when not in use. Figure 2 shows the preparation process of the CMS-*g*-PANI@MWCNTs/Tyrase/GCE.

## 3. Results

### 3.1. Characterization of Nanocomposite Using FTIR and XRD Spectroscopies

**Figure 3:**
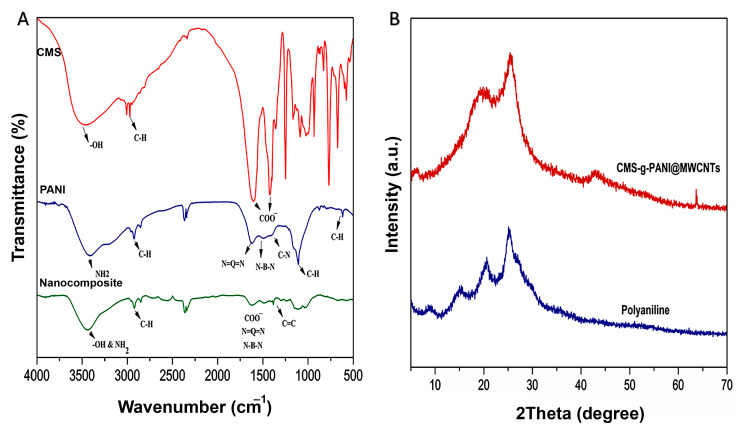
(**A**) The FT-IR spectra of carboxymethyl starch (CMS), polyaniline (PANI), and CMS-*g*-PANI@MWCNTs nanocomposite; (**B**) The XRD patterns of CMS-*g*-PANI@MWCNTs nanocomposite and PANI.

The FTIR spectrum of CMS-*g*-PANI@MWCNTs nanocomposite was analyzed by comparing the spectra of CMS and PANI (Figure 3A). In the FTIR spectrum of CMS, three absorption bands around 1081 cm^−1^, 1156 cm^−1^, and 1020 cm^−1^ were attributed to the C-O stretching vibration of C-O-H and C-O-C groups, respectively [25]. The absorption peak at about 2930 cm^−1^ was attributed to the C-H stretching vibration of pyranoid rings in the starch [26]. The observed broadband at 3490 cm^−1^ was ascribed to the OH stretching vibrations [23]. Two new sharp peaks that appeared at 1420 cm^−1^ and 1602 cm^−1^ were related to the symmetrical and unsymmetrical stretching vibrations of COO^−^, respectively [23]. In the spectrum of PANI, a wide band at 3402 cm^−1^ results from N-H stretching vibrations in the aromatic amine [27]. Two peaks around 1560 cm^−1^ and 1480 cm^−1^ are due to the stretching vibration of C=N and C=C in the benzenoid and quinoid moieties in the PANI chains [28]. In the spectrum of CMS-*g*-PANI@MWCNTs, interactions between OH and N-H groups from the CMS and PANI, respectively, caused a change in intensity and peak position at 3432 cm^−1^ [26]. Similar peaks also observed at 1601 cm^−1^ and 1420 cm^−1^ can be attributed to CMS and around 1560 cm^−1^, and 1481 cm^−1^ may be assigned to PANI with a slight decrease in absorption. The band at 1650 cm^−1^ is attributed to the stretching of C=C, which exhibits the graphite structure of MWCNTs [29].

Following the successful formation of the nanocomposite, according to the FTIR results, the structure and crystallinity of the CMS-*g*-PANI@MWCNTs, CMS, and PANI were studied by the X-ray diffraction pattern (Figure 3B). X-ray diffraction patterns of CMS display an amorphous structure that can be attributed to the modification of the pure starch by carboxyl groups [23]. Pure polyaniline comparatively sharp peaks show a typical semicrystalline pattern [26]. The diffraction peak at 2θ = 25.059° in the CMS-*g*-PANI@MWCNTs pattern corresponds to the PANI and CMS. Peaks at 2θ = 15.32° and 21.01° were also related to emeraldine PANI [23,30]. In the pathway, copolymerization does not observe special intermolecular interaction between carbon nanotubes and copolymers. According to observed patterns from CMS and PANI, the amorphous structure of CMS-*g*-PANI@MWCNTs nanocomposite can be justified.

### 3.2. FE-SEM Images for Surface Morphological Characterizations

**Figure 4:**
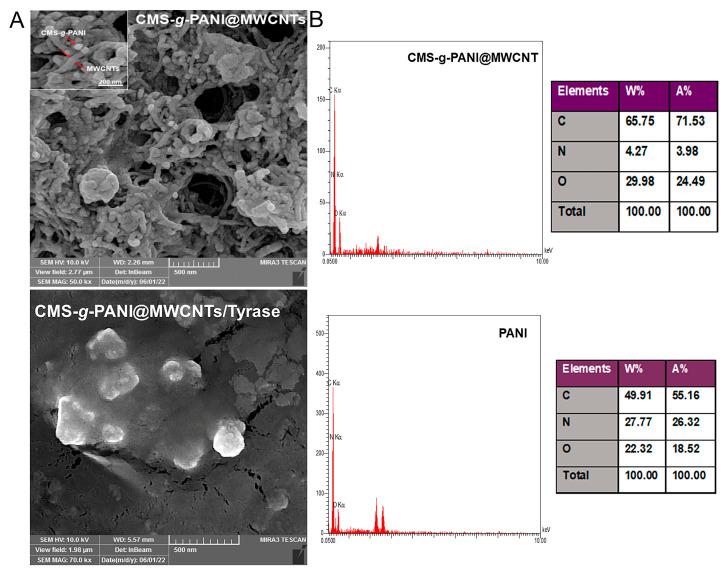
The FE-SEM images (**A**) of CMS-*g*-PANI@MWCNTs and CMS-*g*-PANI@MWCNTs/Tyrase/GCE modified electrode. EDS (**B**) spectra and tabulated data of CMS-*g*-PANI@MWCNTs nanocomposite and PANI.

According to the FE-SEM images of the modified electrode surface with CMS-*g*-PANI@MWCNTs nanocomposite (Figure 4A), it can be seen that the carbon nanotubes are irregularly distributed on the accumulated carboxymethyl starch-*graft*-polyaniline surface [27]. Surface morphology for CMS-*g*-PANI@MWCNTs/Tyrase/GCE modified electrode shows a monotonic surface. The nanocomposite porous structure caused the enzyme uniformly entrapped on the surface [31]. The chemical composition of CMS-*g*-PANI@MWCNTs nanocomposite was evaluated by EDS and compared with polyaniline chemical composition (Figure 4B). The existence of increased values of carbon elements in the nanocomposite was attributed to the polyaniline and carbon nanotubes. The nitrogen element, which is characteristic of polyaniline, indicates that the nanocomposite was successfully formed. The existence of oxygen elements in the EDS can attribute to the entrapped APS initiator in the PANI chains in the copolymerization pathway.

### 3.3. Electrochemical Impedance Spectroscopy

**Figure 5:**
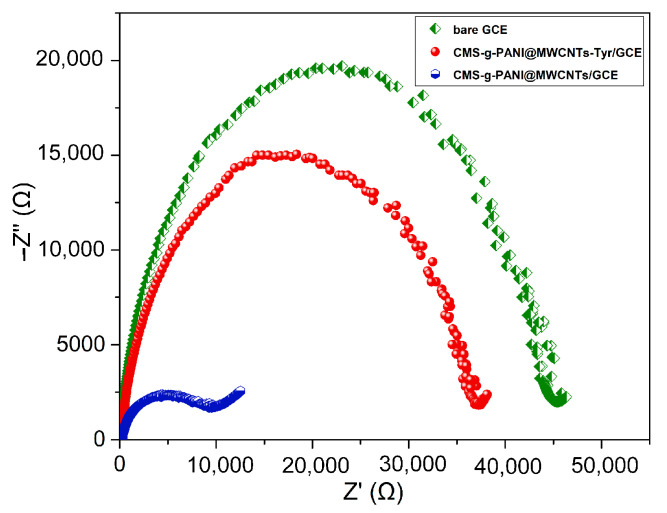
The bare GCE, CMS-*g*-PANI@MWCNTs/GCE and CMS-*g*-PANI@MWCNTs/Tyrase/GCE Nyquist plots in 5.0 mM K_3_[Fe(CN)_6_]/K_4_[Fe(CN)_6_] redox probe and 0.1 M KCl as the supporting electrolyte. AC and DC amplitude potentials of 5 mV; frequency range: 0.1–50 kHz.

To describe the interfacial characteristics of the modified electrodes and electron transfer phenomena, the bare electrode impedance spectra and the electrode modified with nanocomposite (CMS-*g*-PANI@MWCNTs) in the presence and absence of the Tyrase were measured using 5 mL phosphate buffer 0.1 M at pH 6.8 and 5 mM [Fe(CN)6]^3−^/^4−^ redox. The value of surface resistance (Rct) for electron transfer can be obtained from the semicircle diameter in the Nyquist plots, which changes with the presence of different materials on the electrode surface. In Figure 5, the Nyquist plot for the bare electrode (green curve) has a semicircle with a larger diameter than the other two semicircles, which means a higher charge-transfer resistance and a decrease of the current at the electrode/electrolyte shared surface. The diameter of the semicircle is significantly reduced in the impedance spectrum of the modified electrode by nanocomposite (blue circle), which means acceleration of the electron transfer by electroactive nanocomposite as the electrode/electrolyte interface. The increase in the diameter of the Nyquist semicircle of CMS-*g*-PANI@MWCNTs/Tyrase/GCE indicates the stabilization of the Tyrase on the electrode surface and the blocking effects of the enzyme on charge transfer [32] due to the participation of electrons in enzymatic redox reactions.

### 3.4. Electrochemical Features of CMS-g-PANI@MWCNTs/Tyrase/GCE

#### 3.4.1. CV Studies of CMS-*g*-PANI@MWCNTs/Tyrase/GCE Film

**Figure 6:**
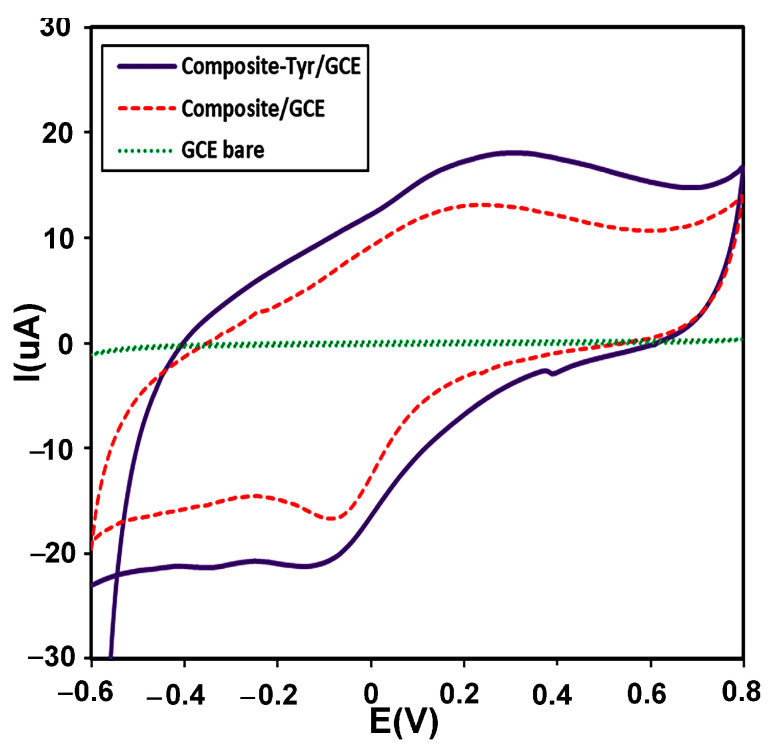
CVs of the bare electrode (green curve), the modified electrode with a CMS-*g*-PANI@MWCNTs nanocomposite (red curve), and the modified electrode with a CMS-*g*-PANI@MWCNTs/Tyrase (Purple curve), in 0.1 M potassium phosphate buffer (pH 6.8) at a scanning speed of 100 mVs^−1^.

CV is usually used as a selection method for the primary investigation of electrochemical systems. The electrochemical behavior of the modified electrode with the CMS-*g*-PANI@MWCNTs/Tyrase film compared to the CMS-*g*-PANI@MWCNTs modified electrode and bare electrode were studied by CV in 5 mL PBS 0.1 M (pH 6.8) at a scan rate of 100 mVs^−1^ vs. Ag/AgCl. As seen in Figure 6, CMS-*g*-PANI@MWCNTs/Tyrase CV shows a pair of enhanced redox peaks than the CMS-*g*-PANI@MWCNTs film at potentials about +0.25 and −0.1 V at a scan rate of 100 mV s^−1^, while a redox peak was not observed in the bare electrode voltammogram. The wide cathodic and anodic peaks in the CMS-*g*-PANI@MWCNTs voltammogram can be attributed to polyaniline [33], the current intensity of which increases after adding Tyrase on the modified electrode. The cathode current ratio to the anodic current (Ip_c_/Ip_a_) is close to one, indicating the reversible electron transfer between immobilized Tyrase and the electrode surface. The presence of polyaniline as a conductive component in the nanocomposite has a great effect on the electron current by increasing the conductivity. The formal potential (E°′) for quasi-reversible peaks of the CMS-*g*-PANI@MWCNTs/Tyrase film was calculated at 0.1 V. This value is attributed to Cu (II) ions couple present in the active site of Tyrase according to various sources and methods of immobilization [34]. These results confirm the immobilized Tyrase redox activity on the nanocomposite. It seems a biocompatible microenvironment will be provided by carboxymethyl starch in the nanocomposite for Tyrase absorption due to electrostatic interactions between carboxyl groups and functional groups of amine on the Tyrase [18]. Thus, carboxymethyl starch and carbon nanotube (due to the high surface-to-volume ratio) improve more loading of Tyrase on the surface of GCE.

#### 3.4.2. pH Effect

**Figure 7:**
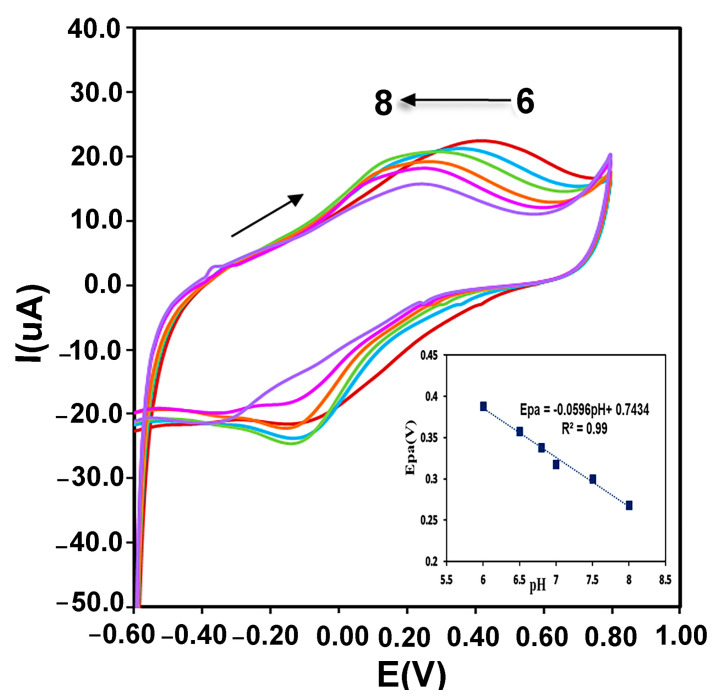
The pH effect on the redox behavior of CMS-*g*-PANI@MWCNTs/Tyrase/GCE at different pH (from 6.0 to 8.0) in 0.1 M PBS solution. Inset: plots of anodic potential vs. pH.

Immobilized enzyme redox reactions on the surface of the electrode can be affected by the protons of electrolyte. Therefore, the effect of electrolyte pH changes on the CMS-*g*-PANI@MWCNTs/Tyrase/GCE biosensor response was evaluated in 0.1 M phosphate buffers at a range of 6.0 to 8.0 and scan rate of 100 mVs^−1^. According to the recorded voltammograms in Figure 7, the potential of the anodic peak changes linearly shifted to more negative values with increasing pH of this range. A linear regression equation of Epa = −0.0596 pH + 0.7434, R^2^ = 0.99 was obtained between pH and oxidation peak potential which the slope of anodic potential vs. pH (−59.6 mV/pH) was equal to the theoretical slope value in the Nernst equation (−59 mV/pH), which shows the ratio of involved electrons and protons in the reaction is 1:1 [35]. As a result, the environment pH and the amino acids protonation/deprotonation in the enzyme active site play an important role in the immobilized enzyme catalytic activity. Furthermore, the highest intensity of the current is obtained at a pH between 0.6 to 0.7, which corresponds to the optimal pH of Tyrase activity in the literature.

#### 3.4.3. Scan Rate Effect

**Figure 8:**
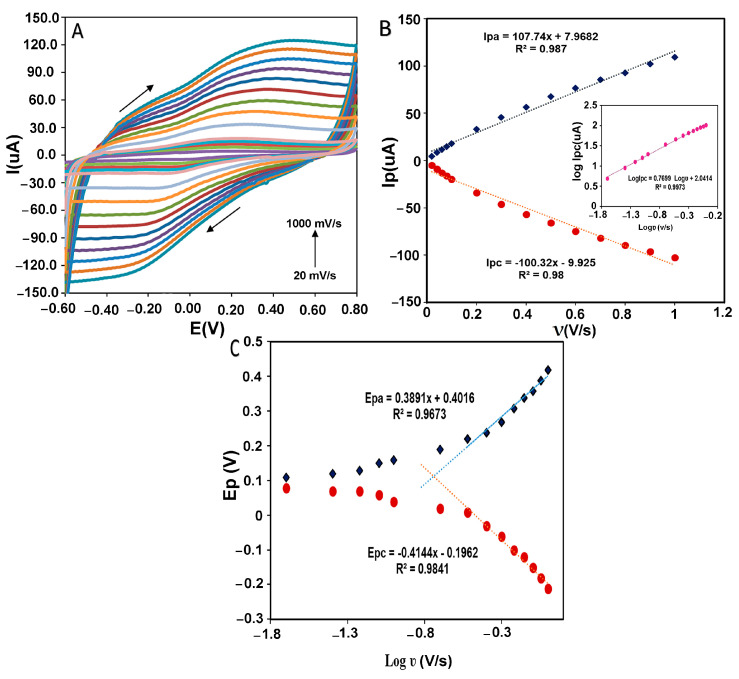
(**A**) CVs of CMS-*g*-PANI@MWCNTs/Tyrase/GCE at varying scan rates in 0.1 mM PBS buffer of pH 6.8. [0.02; 0.04; 0.06; 0.08; 0.1; 0.2; 0.3; 0.4; 0.5; 0.6; 0.7; 0.8; 0.9; 1.0 mV s^−1^]. (**B**) Dependence of anodic and cathodic peak current against the scan rate. Inset B: log I_pc_ vs. log ν. (**C**) Dependence of anodic and cathodic peak potential versus log scan rate from 0.02 to 1.0 mV s^−1^.

The estimation of kinetic parameters gives us information about the direct electron transfer behavior in the designed electrochemical system. Figure 8A shows the peak potential changes in different scan rates from 20 to 1000 mVs^−1^ for CMS-*g*-PANI@MWCNTs/Tyrase/GCE in 5 mL phosphate buffer 0.1 M (pH 6.8). By increasing the scan rate, the anodic and cathodic peak potentials were shifted towards a more positive and negative value, respectively. According to Figure 8B, both the anodic and cathodic peak currents (I_pa_ and I_pc_) were linearly increased with the scan rate change. The regression equation for cathodic and anodic peak currents was: I_pa_(μA) = 107.74x + 7.9682, R^2^ = 0.987 and I_pc_(μA) = −100.32x − 9.925, R^2^ = 0.98, which means the electrode reaction corresponds to a typical surface-controlled electrochemical process, as expected for immobilized systems [36]. The linear slope of log I_pc_ vs. log ν (inset of Figure 8B) was obtained at 0.76 (R^2^ = 0.9973), which is almost close to one, as expected for the electroactive species behavior of the thin layer [37]. The amount of immobilized Tyrase on the electrode surface can be estimated by using the slope of I_pc_ and placing it in the Laviron theory equation (Equation (1)) [38]:

I_pc_ = n^2^F^2^AΓʋ/4RT (1)

where Γ (mol cm^−2^) is the surface coverage of adsorbed Tyrase, A (cm^2^) is the real electrode surface area, and n is the number of electrons transferred in the rate-determining reaction, and the other symbols have their known meanings. Assuming a two-electron transfer in the Tyrase redox system, the surface coverage of the electroactive species was calculated as 8.5 × 10^−10^ mol cm^−2^ and indicated that approximately a thin layer of Tyrase molecules participate in electron transfer with the electrode surface. This result is in agreement with the result of log I_pc_ vs. logν. Figure 8C shows the anodic and cathodic peak potentials vs. the logarithm of scan rate (log ʋ). Based on the Laviron theory, at high scan rates, if the values of “n∆EP > 0.2 V” are obtained experimentally, anodic and cathodic peak potentials have a linear relationship with the logarithm of the scan rates for a quasi-reversible electrochemical system [37]. According to the obtained linear equations as E_pc_ = −0.4144x − 0.1962 (R^2^ = 0.9841) and E_pa_ = 0.3891x + 0.4016 (R^2^ = 0.9673) and using slopes of −2.3RT/αnF and 2.3RT/(1 − α)nF for the cathodic and anodic peak, respectively, charge-transfer coefficient (α) was calculated 0.49, which is close to the calculated value of 0.5 in Laviron computation. The heterogeneous transfer rate constant (k_s_) was then obtained 0.4 ± 0.016 s^−1^ using Equation (2) [37]:

log ks = αlog(1 − α) + (1 − α) − log(RT/nFν) − α(1 − α)(nFΔEP/2.3RT)(2)

Which, in comparison with the values calculated by the silver electrode (0.03 s^−1^) [39] and other Tyrase biosensors (Tyr/MWCNTs/GCE 2.103 × 10^−7^ s^−1^ [40]) (Tyr-AuNPs/BDD 0.032 s^−1^ [41]), is faster. Therefore, it can be said the nanocomposite facilitated electron transfer between the immobilized Tyrase and GCE surface as a conductive matrix by polyaniline and carbon nanotube.

#### 3.4.4. Electrocatalytic Properties of Immobilized tTyrase Using Differential Pulse Voltammetry (DPV)

**Figure 9:**
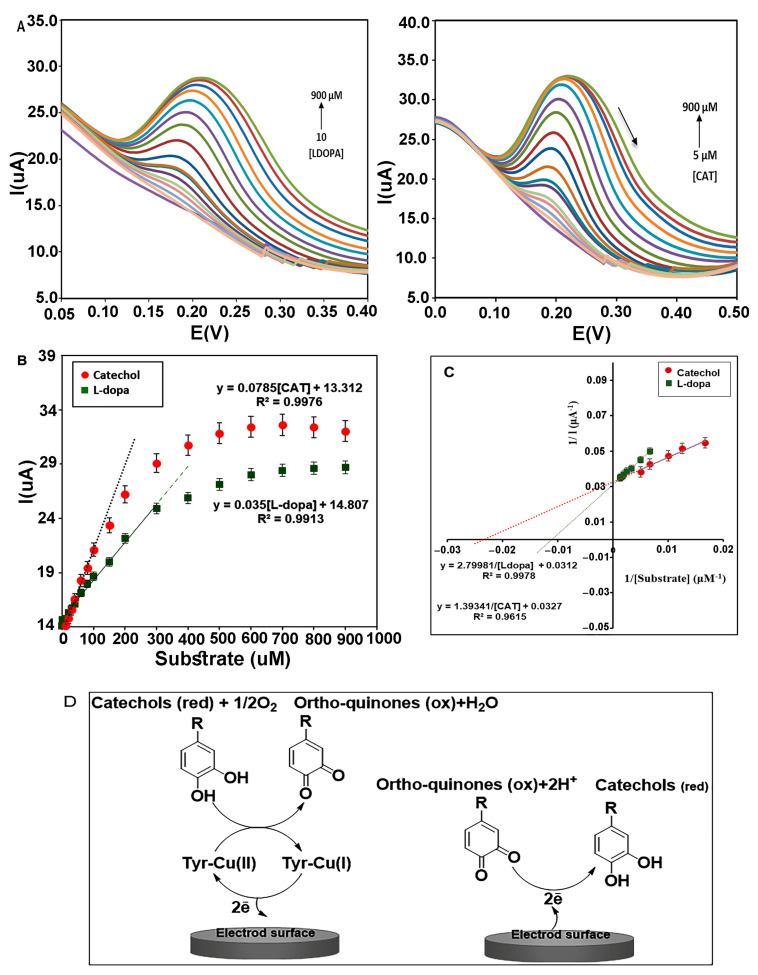
(**A**) DPVs of nanocomposite (CMS-*g*-PANI@MWCNTs) in the presence of L-dopa and catechol in PBS buffer of pH 6.8. (**B**) The Michaelis–Menten diagram of the biosensor for different concentrations of catechol and L-dopa. (**C**) Lineweaver–Burk plots of biosensor for L-dopa and Catechol. (**D**) Electron transfer between Tyrase and the electrode surface during diphenols activity. (Left electrode) Anodic reaction, (Right electrode) Cathodic reaction.

The immobilized enzyme on the electrode surface exists in its primary oxidation form (oxy-tyrosinase) with two copper ions (Cu(II)). By oxidation of diphenols to ortho-quinone and water, it converts to a met-Tyrase while retaining the oxidation state of the active site copper ions [Cu(II)]. Following an increase in the concentration of the substrate, diphenols can bind to both oxy and met-oxy forms and produce more ortho-quinone, and active site copper ions were reduced to Cu(I) and give deoxy-tyrosinase. During these exchanges, two electrons are transferred to the electrode surface (Figure 9D), and, thus, the anodic peak current gradually increases. Ortho-quinones can be reduced to catechol in a reversible reaction by receiving electrons from the electrode surface using applied potential, and, thus, the cathodic peak current is gradually increased. However, an increase in the cathodic peak was not observed in these investigations. Perhaps it can be said that during the catecholic cycle, a catechol can be oxidized as a phenol by oxy-Tyrase using a monooxygenase pathway, leading to a reduction of one of the copper atoms to the Cu(0) state and diffusing out of the active site, with the enzyme finally inactivated [42].

The electrocatalytic behavior of the immobilized enzyme on the film of CMS-*g*-PANI@MWCNTs was analyzed using two specific substrates of Tyrase (catechol and L-dopa), in 5 mL potassium phosphate buffer 0.1 M (pH 6.8) by DPV technique (*n* = 3) (Figure 9A). Voltammetric peak currents measured are directly related to the concentration of phenolic compounds, so that increasing the concentration of L-dopa, the current intensity of the anodic peak linearly increases in the range of 10 to 300 μM (in the potential of 0.2 V) with a linear regression equation *I_pa_* = 0.035[L-dopa] + 14.807 (*R*^2^ = 0.9913). With the further increase in the L-dopa concentration, the intensity of the current increases slowly until it stops at high concentrations (Figure 9B).

The slope of the obtained line in the Michaelis–Menten plot indicates the sensitivity of the biosensor to L-dopa (1.11 μA μΜ^−1^ cm^−2^). According to the equation LOD=3.3 × (Standard deviation of the regression line(σ))/Slope(S) [43], the lowest detection limit was obtained 32 ± 0.38 μΜ. The Lineweaver–Burk diagram was drawn following Michaelis–Menten’s plot to measure the maximum current) I_max_) and the apparent Michaelis–Menten constant (K_m_^app^) for a saturated enzyme with the substrate. According to the linear regression equation, “y = 2.7998x + 0.0312”(R^2^ = 0.9978), the values of K_m_^app^ and I_max_ for different concentrations of L-dopa (150, 200, 300, 400, 500, 600, 700, 800 μM) were 86 μΜ and 32 μA, respectively (Figure 9C). All laboratory analyses were similarly performed for catechol. A linear regression response of the anodic peak was obtained as Ipa=0.0785 [CAT]+13.312 (*R*^2^ = 0.9976) in the range of 5 to 300 μM. The sensitivity and LOD were obtained at 2.4 μA μΜ^−1^ cm^−2^ and 25 ± 0.66 μΜ, respectively (Figure 9C). The K_m_^app^ and I_max_ values for various concentrations of catechol (60, 80, 100, 150, 200 μM) were estimated to be 42 μΜ and 31 μA from the linear equation y=1.3934x+0.0327 (R^2^ = 0.9615), respectively (Figure 9C). These results are more favorable compared to other reported PANI/Tyr-based biosensors using catechol as the substrate. For example, PANI– glutaraldehyde–polyphenol oxidase biosensor (I_max_ 9.44 μA and K_m_^app^ 117 μΜ [44]), PANI-CA biosensor (I_max_ 3.08 μA and K_m_^app^ 77.52 μΜ [45]), PANI–PPO film (I_max_ 0.62 μA and K_m_^app^ 146 μΜ [46]), and PANI(T)–PPO (K_m_^app^ 85.44 μΜ [31]), whereas it is higher than that for the PANI/Tyr-SWCNTs/GCE (K_m_^app^ 24.71 μΜ) [47]. According to the inherent characteristic of the Michaelis–Menten constant (k_m_), lowering this kinetic parameter is an indication of a stronger affinity between enzyme and substrate [48]. The overlap of Michaelis–Menten diagrams illustrate the steeper slope for catechol than L-dopa, which indicates more sensitivity of the biosensor to catechol. Comparing the K_m_^app^ for the two substrates can show more affinity of Tyrase for catechol. As mentioned in the articles, the catalytic power (V_max_/K_m_) of Tyrase increases with a decrease in the size of the side chain in the aromatic ring of its substrates [49].

#### 3.4.5. Selectivity of the Biosensor

**Figure 10:**
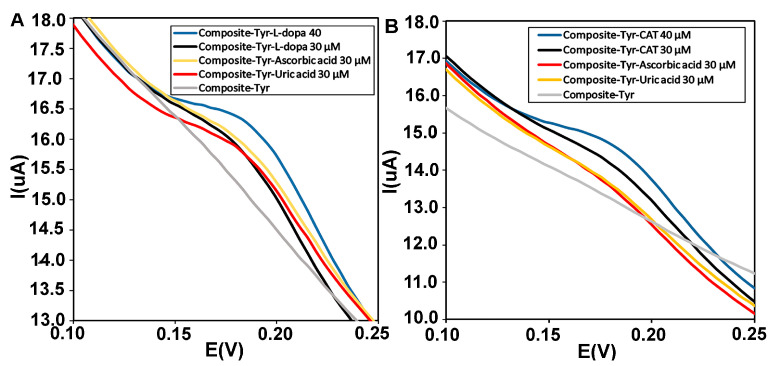
(**A**) Differential pulse voltammograms of the CMS-*g*-PANI@MWCNTs/Tyrase/GCE biosensor in the presence of similar concentrations (30 μΜ) of L-dopa (black curve), uric acid (orange curve) and ascorbic acid (red curve) and (**B**) catechol (black), uric acid (orange), and ascorbic acid (red) in 0.1 M phosphate buffer, pH 6.8.

Electroactive biomolecules such as ascorbic acid (AA) and uric acid (UA) with oxidation potentials similar to catechol and L-dopa can lead to overlapping voltammetric responses in electrochemical biosensors. Therefore, selective and sensitive detection of catechol and L-dopa in the presence of interfering species is necessary [50]. The anti-interference ability of the CMS-*g*-PANI@MWCNTs/Tyrase/GCE biosensor was investigated by DPV using 5 mL PBS 0.1M (pH = 6.8). During sensing by adding similar concentrations of uric acid, ascorbic acid, and L-dopa (30 μΜ), the biosensor showed overlapped current intensity in the range of L-dopa’s oxidative potential, in the next step with adding L-dopa concentration (40 μΜ), the enhanced voltammetric response was not affected by interfering reductive species (Figure 10A). In Figure 10B, obtained voltammograms of 30 μΜ of uric acid and ascorbic acid compound showed no significant changes, while the peak current intensity related to concentrations of catechol significantly increases.

#### 3.4.6. The Stability of the Biosensor

**Figure 11:**
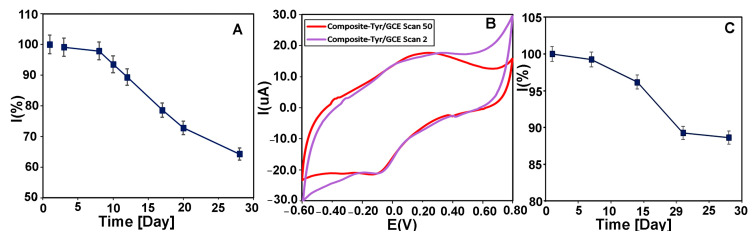
(**A**) The long-term stability of CMS-*g*-PANI@MWCNTs/Tyrase/GCE biosensor by measuring its response current to 20 μM CAT during 28 days in 0.1 M PBS (pH = 6.8) at a scan rate of 100 mV s^−1^. (**B**) CVs of CMS-*g*-PANI@MWCNTs/Tyrase film on glassy carbon electrode in the 2nd cycle (purple curve) and the 50th cycle (red curve). (**C**) Time stability diagram of CMS-*g*-PANI@MWCNTs/Tyrase/GCE biosensor during 28 days.

The functional stability of the biosensor was investigated by measuring the CMS-*g*-PANI@MWCNTs/Tyrase/GCE current response with 20 μM of L-dopa and catechol buffer in 5 mL PBS electrolyte 0.1 M (pH 6.8) at a scan rate of 100 mVs^−1^ using CV every day. The biosensor retained 64% of its initial catalytic power with a decrease in the peak height after 28 days (Figure 11A), while the peak potential remained unchanged. It was reported that the biosensor of Tyr-nAu-GCE remained stable performance for only 18 days [51]. In another work, the Tyr/PO4-Ppy-Pt biosensor maintained 70% of stability only after 10 days [52]. In a similar work, the PANI-Triton X-100/Tyrase biosensor retained 65% of stability after 25 days [31]. These results show the relative stability of our biosensor compared to other reported Tyrase-based biosensors. Figure 11B also shows the voltammograms of the CMS-*g*-PANI@MWCNTs/Tyrase film at a scan rate of 100 mVs^−1^ in the second (purple curve) and 50th (red curve) cycles. As can be seen, after the 50th cycle, not only the intensity of the peaks has not decreased, but it has also become relatively stronger. This shows the functional stability of the CMS-*g*-PANI@MWCNTs/Tyrase film-based electrode.

For evaluation of the storage stability, CMS-*g*-PANI@MWCNTs/Tyrase/GCE electrode was kept at 4.0 °C for 28 days, and every seven days, its electrochemical response was assessed in the absence of substrates. After this time, the biosensor maintained 88% of its initial current response (Figure 11C).

## 4. Conclusions

In this study, a carboxymethyl starch-graft-polyaniline@carbon nanotube (CMS-*g*-PANI@MWCNTs) nanocomposite was prepared by an in situ copolymerization method and used as a substrate for Tyrase immobilization. The electrochemical behavior of immobilized Tyrase on the nanocomposite was then evaluated for the detection of catechol and L-dopa compounds. The FTIR and EDX analyses showed that the CMS-*g*-PANI@MWCNTs nanocomposite was fabricated successfully. The presence of -OH, -COO^−^, and -NH_2_ in the chemical structure of nanocomposite led to good electrostatic interactions with the Tyrase enzyme. The electrocatalytic behavior of the immobilized enzyme on the film of CMS-*g*-PANI@MWCNTs by CV and DPV techniques showed an improvement of electron transport between the enzyme and the electrode surface (Ks = 0.4 s^−1^) due to the synergistic effect of the constituent elements of the nanocomposite. The designed biosensor displayed linearity towards catechol and L-dopa in the concentration range of 5–100 and 10–300 μM with a sensitivity of 2.4 and 1.11 μA μΜ^−1^ cm^−2^ and LOD 25 and 30 μM, respectively. In addition, the CMS-*g*-PANI@MWCNTs/Tyrase/GCE biosensor was better than previously reported biosensors with a K_m_ of 86 and 42 μM for L-dopa and catechol, respectively. According to the analytical performance of the CMS-*g*-PANI@MWCNTs/Tyrase/GCE modified electrode with high functional and storage stability (64% and 88% during 28 days, respectively), it can be concluded that the development of combined methods can be used in the construction of redox reactions-based biosensors and catechol assay in real samples.

## Data Availability

Data available on request.
